# Gallstones and the risk of biliary tract cancer: a population-based study in China

**DOI:** 10.1038/sj.bjc.6604047

**Published:** 2007-11-13

**Authors:** A W Hsing, Y-T Gao, T-Q Han, A Rashid, L C Sakoda, B-S Wang, M-C Shen, B-H Zhang, S Niwa, J Chen, J F Fraumeni

**Affiliations:** 1 Division of Cancer Epidemiology and Genetics, National Cancer Institute, Bethesda, MD, 20852-7234, USA; 2 Shanghai Cancer Institute, Shanghai, China; 3 Ruijin Hospital, Shanghai Second Medical University, Shanghai, China; 4 Department of Pathology, University of Texas MD Anderson Cancer Center, Houston, TX 77030, USA; 5 Department of Epidemiology, University of Washington, Seattle, WA 98195; 6 Zhongshan Hospital, Fudan University, Shanghai, China; 7 Shanghai Tumor Hospital, Shanghai, China; 8 Institute of Oriental Hepatobiliary Surgery, Second Military Medical University, Shanghai, China; 9 Westat Inc., Rockville, MD, USA; 10 University of Pennsylvania, Philadelphia, PA, USA

**Keywords:** biliary tract cancer, gallstones, cholecystitis, China

## Abstract

We conducted a population-based study of 627 patients with biliary tract cancers (368 of gallbladder, 191 bile duct, and 68 ampulla of Vater), 1037 with biliary stones, and 959 healthy controls randomly selected from the Shanghai population, all personally interviewed. Gallstone status was based on information from self-reports, imaging procedures, surgical notes, and medical records. Among controls, a transabdominal ultrasound was performed to detect asymptomatic gallstones. Gallstones removed from cancer cases and gallstone patients were classified by size, weight, colour, pattern, and content of cholesterol, bilirubin, and bile acids. Of the cancer patients, 69% had gallstones compared with 23% of the population controls. Compared with subjects without gallstones, odds ratios associated with gallstones were 23.8 (95% confidence interval (CI), 17.0–33.4), 8.0 (95% CI 5.6–11.4), and 4.2 (95% CI 2.5–7.0) for cancers of the gallbladder, extrahepatic bile ducts, and ampulla of Vater, respectively, persisting when restricted to those with gallstones at least 10 years prior to cancer. Biliary cancer risks were higher among subjects with both gallstones and self-reported cholecystitis, particularly for gallbladder cancer (OR=34.3, 95% CI 19.9–59.2). Subjects with bile duct cancer were more likely to have pigment stones, and with gallbladder cancer to have cholesterol stones (*P*<0.001). Gallstone weight in gallbladder cancer was significantly higher than in gallstone patients (4.9 *vs* 2.8 grams; *P*=0.001). We estimate that in Shanghai 80% (95% CI 75–84%), 59% (56–61%), and 41% (29–59%) of gallbladder, bile duct, and ampulla of Vater cancers, respectively, could be attributed to gallstones.

Cancers of the biliary tract encompass those arising from the gallbladder, extrahepatic bile ducts, and ampulla of Vater. Biliary cancer is relatively uncommon in most parts of the world, although high-risk populations and upward incidence trends have been reported in certain areas (Hsing *et al*, 1998, 2006). Although gallstones are a well-documented risk factor for gallbladder cancer (Diehl, 1983, 1991; Zatonski *et al*, 1997; Lazcano-Ponce *et al*, 2001; Hsing *et al*, 2006), their role in cancers of the extrahepatic bile duct and ampulla of Vater is less established.

From 1972 to 1994, biliary tract cancer was the most rapidly rising malignancy in Shanghai (Hsing *et al*, 1998), involving all three subsites, both sexes, and all age groups. We therefore conducted a population-based case–control study there in 1997 to 2001 to assess the role of gallstones for each biliary cancer subsite.

## MATERIALS AND METHODS

### Cancer cases

The study was approved by the Institutional Review Boards at both the National Cancer Institute and the Shanghai Cancer Institute. Informed consent was obtained for all subjects. Patients with primary biliary tract cancer (ICD9 156), newly diagnosed between June 1997 and May 2001, were identified through a rapid-reporting system established between the Shanghai Cancer Institute and 42 collaborating hospitals in 10 urban districts of Shanghai (henceforth referred to as urban Shanghai), which captured over 95% of relevant cases. Cancer cases were permanent residents of urban Shanghai without a previous non-skin cancer. A total of 627 biliary cancers were enrolled, representing over 90% of the eligible cases.

### Pathology and clinical review

The cancer site was determined from the surgical/pathology report of the local hospital and confirmed by reviewing radiologic data, including magnetic resonance imaging, ultrasonography, computer tomography, and endoscopic retrograde cholangiopancreatography and histopathology. Because biliary cancer is usually diagnosed at a fairly late stage, many tumours were unresectable, and only 458 (68.8%) cases had histological specimens available for review. Confirmation was both clinical and through independent slide review by local pathologists and also by a panel of five leading Shanghai pathologists and one from the US (AR). A consensus review was carried out to resolve any discrepancy. Over 74% of gallbladder and 57% of bile duct cancer cases were confirmed by histology. Whenever possible, staging was evaluated based on the tumour–node–metastasis system of the American Joint Committee on Cancer.

For a total of 185 (29%) cases without pathological tissue, mostly due to unresectable tumours, imaging data as well as clinical and operative reports were referred to the clinical review panel (four leading gastrointestinal surgeons and a pathologist). A total of nine cases with neither tissue nor clinical data for confirmation were excluded from the study.

### Gallstone cases

A total of 1037 patients with biliary stones undergoing cholecystectomy or medical treatment at the same hospital as the index case were selected and matched by gender and age (within 7 years) (1) to assess risk factors specific to cancer and (2) to collect such samples as gallstones, bile, and snap-frozen tissue for biochemical and molecular analyses. Over 95% of eligible stone cases participated. Similar clinical and histopathological assessments of the biliary tract were carried out for all cases with stones.

### Population controls

A total of 959 healthy subjects, without a history of cancer, were randomly selected from the 6.5 million permanent residents of Shanghai as population controls, using the personal registry cards of all adults over age 18 in urban Shanghai. They were frequency-matched to the expected age distribution (5-year category) of the cancer cases. Controls with a history of non-skin cancer were ineligible: over 83% of eligible controls participated.

### Interviews

In-person interviews were conducted with all study subjects, using a structured questionnaire, by trained interviewers on potential risk factors, including smoking, alcohol, body size, diet, reproductive history, family history of cancer, medical history, and physical activity. Detailed information was obtained on biliary diseases, including a history of gallstones, date of diagnosis and treatment, inflammatory conditions, and surgical procedures. Each interview was taped, reviewed, and verified by a supervisor. In addition, 5% of the controls were randomly selected for a second interview within 3 months after the original interview to assess reproducibility. Concordance between the two interviews was over 95%.

### Gallstone status

Great care was taken to ensure the validity of the classification of gallstone status among study subjects. For cancer cases, in addition to self-reports of a gallstone history, operative reports and imaging data were used; details of gallstone location at cancer diagnosis were collected. Similar procedures were used to confirm gallstone status in the 1037 gallstone patients. For controls, in addition to a self-reported gallstone history, transabdominal ultrasound was performed to validate gallstone status and to identify silent gallstones. Evidence of biliary sludge based on ultrasound examination was also recorded, although sludge alone was not classified as gallstones. One hundred and twenty (13%) controls did not participate in ultrasound examination; their gallstone status was determined solely by self-reports.

Biological samples, including overnight fasting blood, gallstones, and tissue, were collected from study subjects whenever possible. Information on number, size, colour, and type of gallstones was obtained from surgical and pathology reports and medical records, when available; weight was based on the collected stones. Gallstones were successfully collected from 51 cancer patients and 520 gallstone patients for morphological and biochemical classification. Based on the surface and cross-section of the largest stone, six colour categories were formed: white, light yellow, dark yellow, light brown, dark brown, and black.

### Chemical classification of gallstones

The largest stone was washed by saline and then ground into powder and analysed for cholesterol, bilirubin, and total and specific bile acids, such as cholic, chenodeoxycholic, deoxycholic, glycocholic, glychenodeoxycholic, and glycodeoxycholic acids (Oda *et al*, 1975; Roda *et al*, 1975; Fossati *et al*, 1989; Wang *et al*, 1990). The cholesterol and bile acid content was measured by high-performance liquid chromatography, while bilirubin concentration was measured by enzymatic methods (Roda *et al*, 1975; Han *et al*, 1998). The collected gallstones were dissolved by adding 10 mg of dry gallstone powder in 10 ml dimethylsulphoxide, and were vortexed rigorously for complete dissolving. After addition of sodium tartrate, the OD value at 600 nm was measured on the Beckman ultraviolet spectrophotometer. Cholesterol concentration was estimated as: bilirubin (mg per 100 mg)=10 × OD (Sample)/OD (standard reference). Cholesterol stones were defined as stones whose cholesterol content exceeded 50%.

### Statistical analysis

Unconditional logistic regression was used to compute odds ratios (ORs) and 95% confidence intervals (CIs) for each cancer subsite associated with gallstones (yes/no) separately, adjusting for age (<55, 55–64, ⩾65), sex, and educational level (none or primary, junior middle, senior middle, or some college). Gallbladder cancer patients were compared with population controls with an intact gallbladder (without cholecystectomy), while patients with bile duct or ampullary cancer were compared with control subjects, since cholecystectomy does not involve the bile duct or ampulla of Vater. Sex-specific analyses were performed, since gallstones and gallbladder cancer are more common in women. Covariates, including cigarette smoking, alcohol use, education, and history of diabetes or hypertension, were evaluated as potential confounding variables but excluded from the final regression models since they did not appreciably change the risk estimates. Joint effects on biliary cancers of gallstones and several of their risk factors, including diabetes, body mass index (BMI, weight (kg)/height (m)^2^), chronic cholecystitis, and pancreatitis, were examined by stratified analysis. A cross-product term was included in the regression model to evaluate the significance of interactive effects. All tests were two-sided, with *P*<0.05 defined as statistically significant. Attributable risk was calculated using the Epitome program, using the standard formula PAR=Pe(RR−1)/(1+Pe(RR−1), where Pe is the prevalence of gallstones among controls and ORs the estimate of relative risk (RR) (Rothman and Greenland, 1998).

## RESULTS

A total of 627 biliary cancer patients (368 gallbladder, 191 bile duct, and 68 ampulla of Vater), 1037 stone patients (774 gallbladder and 263 bile duct), and 959 healthy controls were included. Most cancers were diagnosed at an advanced stage: 70, 61, and 44 of gallbladder, bile duct, and ampulla of Vater cancers, respectively, were diagnosed at stage III or IV. Of the tumours with histopathological information, over 90% were adenocarcinomas. Cancers diagnosed incidentally during surgery for gallstones accounted for 26.1, 14.1, and 8.8% of the patients with gallbladder, bile duct, and ampulla of Vater cancers, respectively. Of the 191 extrahepatic bile duct cancers, over half (60%) were in the upper third (including the cystic duct), 6% in the middle third, 10% in bile duct not otherwise specified, 18% in the lower third, and 6% in other or multiple parts. Among gallstone patients, 8.1% had intestinal metaplasia while 5.3% had dysplasia/carcinoma *in situ*.

Table 1 shows selected characteristics of cases and controls with age (due to matching) similar in cancer cases and controls. Those with biliary stones and no cancer tended to be younger than cancer cases. Gallbladder cancer was more common in women than men, while slightly more men had cancers of extrahepatic bile ducts and ampulla of Vater. Relative to controls, gallbladder cancer patients had a lower education level, were less likely to smoke or drink alcohol, more likely to have a history of diabetes, and had a higher BMI.

Table 2 shows ORs for biliary tract cancer in relation to gallstones. The overall prevalence of gallstones in the 959 controls was 23.4%, with 5.9% reporting a history of cholecystectomy, 11.3% of gallstones, and 6.2% having silent gallstones detected through ultrasound. The 5.5% of controls with biliary sludge or cholesterol crystals detected on abdominal ultrasound were not classified as having gallstones. In contrast, a history of gallstones were found for 83.7, 66.5, and 53% of gallbladder, bile duct, and ampulla of Vater cancer, respectively. The ORs associated with stones were 23.8 (95% CI, 17.0–33.4), 8.0 (95% CI 5.6–11.4), and 4.2 (95% CI 2.5–7.0) for cancers of the gallbladder, extrahepatic bile ducts, and ampulla of Vater, respectively. Further adjustment for smoking, drinking, BMI, tea drinking, aspirin use, and diabetes did not materially change the results. Location of gallstones was recorded for over 73% of the cancer patients with gallstones. For those with gallbladder cancer, 77% of the stones were in the gallbladder, 10% in the gallbladder neck, 9% in both the gallbladder and bile ducts, and 4% in bile ducts alone. The corresponding percentages for bile duct cancer were 68, 7, 11, and 13%, and for ampullary cancer 57, 0, 14, and 28%. The average age for biliary tract cancer cases with gallstones was about 5 years older than that for cases without gallstones (65 *vs* 60, *P*=0.0005).

As shown in Table 3, overall, younger age at gallstone diagnosis or longer duration of stones alone did not modify the 23-fold risk associated with gallstones. The very high ORs for bile duct and ampulla of Vater cancers related to having gallstones for 1 year or less were due to the frequency of incidental tumours shortly after or at the time of stone diagnosis. Similar risk patterns were observed for both men and women (data not shown).

Table 4 shows the combined effects of gallstones and related factors, including chronic cholecystitis, pancreatitis, diabetes, and high BMI, on biliary cancer risk. Since gallstones and cholecystitis may be detected concomitantly with biliary tract cancer, and gallstones may induce cholecystitis/pancreatitis, subjects with cholecystitis or pancreatitis diagnosed prior to 2 years of cancer diagnosis (mostly incidental tumours) or interview were excluded from the analysis. Subjects with both gallstones and (self-reported) chronic cholecystitis had a markedly increased risk of biliary cancer, especially of the gallbladder (OR=34.3; 95% CI 19.9–59.2), although the interaction between gallstones and the cholecystitis was not statistically significant. Similarly, subjects with both gallstones and diabetes had a higher risk of gallbladder cancer (OR=30.7, 95% CI 16.7–56.5). BMI and pancreatitis did not appear to modify the effect, and even among those with a low BMI (<23 kg/m^2^); gallstones were a significant risk factor for all three subsites.

Supplementary Information (see website) shows the morphological characteristics of gallstones for 249 biliary cancer patients (196 gallbladder, 53 bile duct) and 892 stone patients (668 gallbladder, 224 bile duct) as well as the biochemical composition of gallstones in 530 subjects with stones alone (358 gallbladder, 162 bile duct), 41 with gallbladder cancer, and 10 with bile duct cancer. Significant differences by subsite were seen in type, number, and weight, with pigment stones being more common in the bile duct patients or with bile duct cancer, and cholesterol stones in gallbladders patients or with gallbladder cancer. Compared with bile duct cancer, gallbladder cancer patients were more likely to have multiple (72 *vs* 58%, *P*=0.08), larger, and heavier stones. Their stones were also heavier (4.9 *vs* 2.9 g, *P*=0.006) than those of gallstone patients, but no significant differences were seen in the size or number of stones. Patients with bile duct stones or extrahepatic bile duct cancer had higher levels of bile acids and bilirubin in their stones than patients with gallbladder stones or cancer (*P*<0.001).

## DISCUSSION

In this population-based case–control study in Shanghai, we confirmed that gallstones were a strong risk factor for all three subsites of biliary cancer. We estimated that 80% (95% CI 0.75–0.84), 59% (0.50–0.61), and 42% (0.29–0.57) of gallbladder, bile duct, and ampulla of Vater cancers, respectively, in the Shanghai population were attributable to gallstones.

Although gallstones are a risk factor common to all three subsites of biliary cancer, differences in the magnitude of risks and in the prevalence and type of stones were notable. Molecular changes, including somatic mutations (such as *K-ras* and *P16)* and loss of heterozygosity, in these subsites have been described (Rashid *et al*, 2002; Ueki *et al*, 2004; Matsuba *et al*, 2005). Aetiologic heterogeneity is further supported by ethnic and geographic patterns between cancer subsites, with gallbladder cancer incidence especially high in native Americans and in Korea, India, and Eastern Europe, and the incidence of bile duct cancer highest in Japan (Hsing *et al*, 2006).

That cholesterol stones were commoner in gallbladder cancer while pigment stones predominated in bile duct cancer is consistent with the view that gallbladder cancer is more associated with lifestyle factors (diet, obesity, etc.), while bile duct cancer is associated with chronic infection or inflammation (Cetta, 1991). Cholesterol stones are associated with lithogenic bile supersaturated with cholesterol, due to increased hepatic secretion of cholesterol or diminished secretion of bile salts and phospholipids that maintain the solubility of cholesterol (el Zayadi *et al*, 1991). In contrast, pigment stones have a high biliary concentration and are closely linked to cirrhosis, chronic infection, and blood disorders (Swidsinski and Lee, 2001). Cholesterol stones are the commonest type in western population, while pigment stones predominate in developing regions of the world, especially in Asia. However, in China, during the past few decades, there has been an increase in cholesterol stones and a decrease in pigment stones, probably related to increasing obesity and a more westernised diet and lifestyle (Huang and Gu, 1987; Zhu *et al*, 1995; Wang *et al*, 2003).

In our study, the magnitude of the biliary cancer risks associated with gallstones was higher than in most previous studies (Lowenfels *et al*, 1985; Vitetta *et al*, 2000), possibly due to our more comprehensive assessment of gallstone status. Most previous studies were smaller (usually<100), not population-based, and lacked subsite-specific risks. A more pronounced association between gallstones and gallbladder cancer has been reported for women than men but in our study the effect of gallstone was similar in both genders.

The carcinogenic mechanisms in biliary cancer are poorly understood, but they may involve inflammatory changes near stones (Albores-Saavedra *et al*, 1980; Carriaga and Henson, 1995). This is supported by our 34-fold risk associated with gallstones together with (self-reported) chronic cholecystitis, the possibly protective effects of aspirin use (Liu *et al*, 2005), and the association with a stone pro-inflammatory *PTGS* gene variant (Sakoda *et al*, 2006).

Despite their high risk, only about 1% of gallstone patients develop biliary cancer (Maringhini *et al*, 1987), indicating that other factors are involved. Except for cholecystitis, we did not detect interactions with other risk factors, perhaps because of small numbers. Further work is needed to clarify the role of infection as well as genetic and metabolic factors.

Several strengths of this study are notable. First, selection bias was minimal due to population-based design with high case ascertainment and participation. Second, nearly complete confirmation of case status minimised misclassification of cases. Third, the large sample size permitted the investigation of risk factors by anatomic subsite. Fourth, any misclassification of gallstones was minimal, since all cases had detailed clinical data, most controls underwent transabdominal ultrasound examination, and the validity of self-reports of gallstone history was high. Since only 6% of the population controls had silent gallstones, misclassification among the 13% of the controls who did not undergo ultrasound is minimal.

Despite the strong association between gallstones and biliary cancer, we could not establish a clear temporal relationship due to our cross-sectional design. However, in previous studies, age at gallstone diagnosis was usually 10–15 years younger than at cancer diagnosis, and risks were greatest among those with gallstones diagnosed at least 10 years earlier (Grimaldi *et al*, 1993). Despite this being the largest population-based study of biliary cancer to date, there was limited statistical power to evaluate the risk factors for ampulla of Vater cancer and the interactive effect of stones with other factors for all three subsites.

In summary, this population-based study in Shanghai confirmed that gallstones are an important risk factor for all three subsites of biliary cancer, particularly when complicated by chronic cholecystitis.

## Figures and Tables

**Table 1:** Selected characteristics of cases and controls

			**Biliary tract cancer (*n*=267)**			**Biliary stones (*n*=1037)**	
**Characteristics**	**Controls^a^**	**Controls^b^**	**Gallbladder cancer**	**Bile duct cancer**	**Ampulla of Vater cancer**	**Gallbladder stones**	**Bile duct stones**
	***n* (%)**	***n* (%)**	***n* (%)**	***n* (%)**	***n* (%)**	***n* (%)**	***n* (%)**
	959 (100)	902 (100)	368 (100)	191 (100)	68 (100)	774 (100)	263 (100)
*Age (years)*							
<55	128 (13.4)	126 (14.0)	49 (13.3)	30 (15.7)	6 (8.8)	245 (31.6)^*^	62 (23.6)^*^
55–64	269 (28.1)	259 (28.7)	96 (26.1)	47 (24.6)	20 (29.4)	218 (28.2)	80 (30.4)
⩾65	562 (58.6)	517 (57.3)	223 (60.6)	114 (59.7)	42 (61.8)	311 (40.2)	121 (46.0)
							
*Sex*							
Male	373 (38.9)	357 (39.6)	99 (26.9)^*^	99 (51.8)	37 (54.4)	263 (34.0)^*^	127 (48.3)
Female	586 (61.1)	545 (60.4)	269 (73.1)	92 (48.2)	31 (45.6)	511 (66.0)	136 (51.7)
							
*Education*							
None/primary	396 (41.3)	365 (40.5)	198 (53.8)^*^	86 (45.0)	29 (42.7)	221 (28.6)^*^	96 (36.5)
Jr. middle	233 (24.3)	221 (24.5)	79 (21.5)	43 (22.5)	16 (23.5)	220 (28.4)	67 (25.5)
Sr. middle	190 (19.8)	184 (20.4)	50 (13.6)	31 (16.2)	15 (22.1)	194 (25.1)	56 (21.3)
Some college	140 (14.6)	132 (14.6)	41 (11.1)	30 (15.7)	8 (11.8)	139 (18.0)	44 (16.7)
							
*Marital status*							
Married	751 (78.3)	703 (77.9)	284 (77.2)	161 (84.3)	55 (80.9)	664 (85.8)^*^	220 (83.7)
Widowed	175 (18.3)	167 (18.5)	77 (20.9)	27 (14.1)	12 (17.7)	96 (12.4)	38 (14.5)
Divorced/separated	21 (2.2)	20 (2.2)	4 (1.1)	1 (0.5)	1 (1.5)	7 (1.0)	2 (0.8)
Never married	12 (1.3)	12 (1.3)	3 (0.8)	2 (1.1)	0 (0.0)	6 (0.8	3 (1.1)
Smoking (%)	285 (29.7)	269 (29.8)	89 (24.2)^*^	76 (39.8)^*^	30 (44.1)^*^	187 (24.2)^*^	96 (36.5)
Alcohol use (%)	198 (20.7)	186 (20.6)	52 (14.1)^*^	50 (26.2)	15 (22.1)	116 (15.0)^*^	51 (19.4)
Diabetes (%)	78 (8.1)	68 (7.5)	51 (13.9)^*^	20 (10.5)	5 (7.4)	81 (10.5)^*^	30 (11.4)^*^
Hypertension (%)	406 (42.3)	376 (41.7)	138 (37.5)	61 (31.9)^*^	20 (29.4)^*^	267 (34.5)^*^	75 (28.5)^*^
							
*Body mass index*							
<18.5	79 (8.3)	78 (8.7)	17 (4.7)^*^	8 (4.2)	1 (1.5)	29 (3.7)^*^	15 (5.7)^*^
18.5–22.9	404 (42.2)	390 (43.3)	130 (35.6)	86 (45.5)	29 (42.6)	259 (33.5)	91 (34.7)
23.0–24.9	197 (20.6)	184 (20.4)	73 (20.0)	49 (25.9)	15 (22.1)	197 (25.5)	62 (23.7)
⩾25.0	278 (29.0)	249 (27.6)	145 (39.7)	46 (24.3)	23 (33.8)	288 (37.3)	94 (35.9)

^*^χ^2^ test, *P*<0.05.

a Included all population controls. Bile duct cancer and ampulla of Vater cancer were compared with all population controls.

b Excluded population controls with a history of cholecystectomy (*n***=**55) or unknown history (*n***=**2). Gallbladder cancer cases were compared with this group.

**Table 2:** Odds ratios^a^ and 95% confidence intervals for biliary tract cancer in relation to gallstones, Shanghai, China

	**Controls^b^**		**Controls^c^**		**Gallbladder cancer**				**Bile duct cancer**				**Ampulla of Vater cancer**			
**Gallstones**	***n***	**%**	***n***	**%**	***n***	**%**	**OR**	**95% CI**	***n***	**%**	**OR**	**95% CI**	***n***	**%**	**OR**	**95% CI**
	959	100.0	902	100.0	368	100.0			191	100.0			68	100.0		
*Total*																
No	735	76.6	735	81.5	60	16.3	1.0	—	64	33.5	1.0	—	32	47.1	1.0	—
Yes^d^	224	23.4	167	18.5	308	83.7	23.8	17.0–33.4	127	66.5	8.0	5.6–11.4	36	53.0	4.2	2.5–7.0
																
*Males*																
No	313	83.9	313	87.7	24	24.2	1.0	—	40	40.4	1.0	—	20	54.1	1.0	—
Yes^d^	60	16.1	44	12.3	75	75.8	23.7	13.3–42.2	59	59.6	8.2	4.9–13.6	17	46.0	4.6	2.3–9.4
																
*Females*																
No	422	72.0	422	77.4	36	13.4	1.0	—	24	26.1	1.0	—	12	38.7	1.0	—
Yes^d^	164	28.0	123	22.6	233	86.6	24.6	16.1–37.7	68	73.9	7.7	4.6–12.9	19	61.3	3.6	1.7–7.6

a Adjusted for age, sex (if applicable), and education level.

b All population controls. Bile duct cancer and ampulla of Vater cancer were compared with all population controls.

c Excluded population controls with a history of cholecystectomy (*n***=**55) or unknown history (*N***=**2). Gallbladder cancer cases were compared with this group.

d Included 59 silent gallstones.

**Table 3:** Odds ratios^a^ and 95% confidence intervals for biliary tract cancer in relation to age at gallstone diagnosis and duration of stones, Shanghai, China

	**Controls^b^**	**Controls^c^**	**Gallbladder cancer**			**Bile duct cancer**			**Ampulla of Vater cancer**		
	***n***	***n***	***n***	**OR**	**95% CI**	***n***	**OR**	**95% CI**	***n***	**OR**	**95% CI**
Total	959	902	368			191			68		
*Duration of gallstones (years)*											
No gallstones	735	735	60	1.0	—	64	1.0	—	32	1.0	—
⩽1	74	70	138	24.5	16.4–36.5	63	10.2	6.6–15.7	20	6.5	3.5–12.1
2–4	23	17	30	23.6	12.1–45.8	7	3.7	1.5–9.1	2	1.7	0.4–7.7
5–9	47	30	35	14.9	8.5–26.2	11	2.9	1.4–5.9	5	2.4	1.9–6.5
⩾10	80	50	90	23.3	15.0–36.3	37	5.6	3.5–9.0	4	1.1	1.4–3.2
											
*Age at diagnosis of gallstones*											
No gallstones	735	735	60	1.0	—	63	1.0	—	32	1.0	—
<45	28	18	41	25.5	13.6–47.8	16	6.2	3.2–12.2	1	0.8	0.1–6.4
45–59	82	53	82	19.2	12.4–29.7	42	6.0	3.8–9.4	9	2.6	1.2–5.6
⩾60	114	96	173	24.0	16.2–35.6	62	7.2	4.7–11.1	21	4.2	2.3–7.8
											
*Age at diagnosis of gallstones and duration of gallstones*											
No gallstones	735	735	60	1.0	—	64	1.0	—	32	1.0	
<55, ⩽1 year	8	7	12	16.1	5.7–45.3	7	8.8	2.8–27.1	2	8.3	1.4–49.1
<55, 2–5 years	2	2	5	22.1	4.1–120.3	2	9.3	1.3–68.7	1	16.5	1.3–204.3
<55, ⩾6 years	60	38	79	25.6	16.0–38.8	33	6.5	3.9–10.6	4	1.7	0.6–5.1
⩾55, ⩽1 year	66	63	126	25.4	16.7–38.8	56	10.3	6.6–16.2	18	6.8	3.5–12.9
⩾55, 2–5 years	34	26	34	17.7	9.7–32.2	8	2.9	1.3–6.7	2	1.5	0.3–6.8
⩾55, ⩾ 6 years	54	31	37	16.8	9.4–30.0	12	2.8	1.4–5.6	4	1.7	0.6–5.2

a Adjusted for age, sex (if applicable), and education level.

b Included all population controls. Bile duct cancer and ampulla of Vater cancer were compared with all population controls.

c Excluded population controls with a history of cholecystectomy (*n*=55) or unknown history (*N*=2). Gallbladder cancer cases were compared with this group.

**Table 4:** Odds ratios^a^ and 95% confidence intervals for biliary tract cancer in relation to gallstones and other factors, Shanghai, China

			**Biliary tract cancer**								
	**Controls**		**Gallbladder**			**Extrahepatic bile duct**			**Ampulla of Vater**		
	***n* b**	***n* c**	***n***	**OR**	**95% CI**	***n***	**OR**	**95% CI**	***n***	**OR**	**95% CI**
*Gallstones and chronic cholecystitis* d											
No gallstones, no cholecystitis	693	693	47	1.0	—	59	1.0	—	27	1.0	—
Chronic cholecystitis only	32	32	4	2.0	0.7–5.8	2	0.9	0.2–3.9	2	1.8	0.4–8.1
Gallstones only	182	136	185	20.9	14.2–30.7	84	6.5	4.4–9.7	31	5.0	2.8–8.7
Gallstones and chronic cholecystitis	38	29	65	34.3	19.9–59.2	24	9.6	5.2–17.6	3	2.8	0.8–9.8
*P-interaction*			*0.78* ^e^			*0.54*			*0.23*		
											
*Gallstones and pancreatitis*											
No gallstones, no pancreatitis	730	730	59	1.0	—	62	1.0		29	1.0	—
Pancreatitis only	4	4	1	3.0	0.3–28.0	1	2.4	0.2–23.3	0	—	
Gallstones only	214	163	292	23.2	16.5–32.6	116	7.8	5.4–11.3	33	4.4	2.6–7.5
Gallstones and pancreatitis	10	4	6	20.8	5.6–77.6	7	8.7	3.2–24.2	1	2.5	0.3–20.5
*P-interaction*			*0.37*			*0.55*			*0.99*		
											
*Gallstones and BMI*											
No gallstones, BMI <23	399	399	31	1.0	—	36	1.0		15	1.0	
No gallstones, BMI ⩾23	335	335	28	1.1	0.6–1.8	27	0.9	0.6–1.6	17	1.4	0.7–2.8
Gallstones, BMI <23	77	62	115	25.2	15.4–41.2	57	9.8	5.9–16.4	15	5.7	2.6–12.3
Gallstones, BMI ⩾ 23	147	105	191	25.0	15.8–39.3	69	6.6	4.1–10.6	21	4.5	2.2–9.1
*P-interaction*			*0.79*			*0.36*			*0.28*		
											
*Gallstones and diabetes*											
No gallstones, no diabetes	688	688	55	1.0	—	61	1.0	—	31	1.0	—
Diabetes only	47	47	5	1.5	0.6–4.0	3	0.8	0.3–2.8	1	0.5	0.1–3.8
Gallstones only	193	146	262	23.8	16.7–34.0	110	7.9	5.4–11.5	32	4.2	2.4–7.1
Gallstones and diabetes	31	21	46	30.7	16.7–56.5	17	8.0	4.0–15.9	4	3.3	1.1–10.1
*P-interaction*			*0.79*			*0.78*			*0.71*		

a Adjusted for age, sex, and education level.

b Included all population controls. Compared with cancer of the bile duct and ampulla of Vater.

c Excluded population controls with a history of cholecystectomy (*n*=55) or unknown history (*N*=2). Compared with cancer of the gallbladder.

d Excluded subjects with cholecystitis or pancreatitis diagnosed within 2 years of cancer diagnosis or interview.
