# Supplementary material for: Gallstones and the risk of biliary tract cancer: a population-based study in China
Source: Br J Cancer. 2007 Nov 13;97(11):1577–82. doi: 10.1038/sj.bjc.6604047 (PMC2360257; doi:10.1038/sj.bjc.6604047)
Supplement: Supplementary Information [file 6604047x1.doc]

|  | **Appendix A** Morphological and chemical characteristics of gallstones in gallbladder and bile duct cancer and gallstone patients | | | | | | | | | | | | | | | | | | | | | | | | | | | | | | | | |  | |  | |  | | |
| --- | --- | --- | --- | --- | --- | --- | --- | --- | --- | --- | --- | --- | --- | --- | --- | --- | --- | --- | --- | --- | --- | --- | --- | --- | --- | --- | --- | --- | --- | --- | --- | --- | --- | --- | --- | --- | --- | --- | --- | --- |
|  |  | **Stones** | | | | | | | | | | | | | |  | | **Biliary Tract Cancer** | | | | | | | | | | | | | ***p-value^a^*** | | | | | | | | | |
|  |  | **Gallstone Cases** | | | | |  | | | **Bile duct stones** | | | | | |  | | **Gallbladder** | | | | |  | | | **Bile Duct** | | | | | *p1****^b^*** | | | *p2* | | *p3* | | *p4* | | |
|  | Morphological classification | N | % | | |  | | | N | | | % | | |  | | N | | | % | |  | | | N | | | % |  | | | |  | |  | |  | | |  |
|  | Gallstones |  |  | | |  | | |  | | |  | | |  | |  | | |  | |  | | |  | | |  |  | | | |  | |  | |  | | |  |
|  | Type^c^ |  |  | | |  | | |  | | |  | | |  | |  | | |  | |  | | |  | | |  |  | | | |  | |  | |  | | |  |
|  | N | 414 | 100 | | |  | | | 175 | | | 100 | | |  | | 138 | | | 100.0 | |  | | | 36 | | | 100.0 |  | | | |  | |  | |  | | |  |
|  | Cholesterol | 164 | 39.6 | | |  | | | 47 | | | 26.9 | | |  | | 65 | | | 47.1 | |  | | | 13 | | | 36.1 |  | | | |  | |  | |  | | |  |
|  | Pigment | 42 | 10.1 | | |  | | | 43 | | | 24.6 | | |  | | 13 | | | 9.4 | |  | | | 9 | | | 25.0 |  | | | |  | |  | |  | | |  |
|  | Mixed | 208 | 50.2 | | |  | | | 85 | | | 48.6 | | |  | | 60 | | | 43.5 | |  | | | 14 | | | 38.9 |  | | | |  | |  | |  | | |  |
|  |  |  |  | | |  | | |  | | |  | | |  | |  | | |  | |  | | |  | | |  | *<0.0001* | | | | *0.05* | | *0.31* | | *0.47* | | |  |
|  | Color^c^ |  |  | | |  | | |  | | |  | | |  | |  | | |  | |  | | |  | | |  |  | | | |  | |  | |  | | |  |
|  | N | 419 | 100 | | |  | | | 190 | | | 100 | | |  | | 137 | | | 100.0 | |  | | | 35 | | | 100.0 |  | | | |  | |  | |  | | |  |
|  | White | 5 | 1.2 | | |  | | | 1 | | | 1 | | |  | | 4 | | | 2.9 | |  | | | 1 | | | 2.8 |  | | | |  | |  | |  | | |  |
|  | Light yellow | 68 | 16.2 | | |  | | | 40 | | | 21 | | |  | | 25 | | | 18.2 | |  | | | 10 | | | 28.6 |  | | | |  | |  | |  | | |  |
|  | Dark yellow | 56 | 13.4 | | |  | | | 20 | | | 10.5 | | |  | | 14 | | | 10.2 | |  | | | 5 | | | 14.3 |  | | | |  | |  | |  | | |  |
|  | Light brown | 80 | 19.1 | | |  | | | 34 | | | 17.9 | | |  | | 31 | | | 22.6 | |  | | | 6 | | | 17.1 |  | | | |  | |  | |  | | |  |
|  | Dark brown | 156 | 37.2 | | |  | | | 67 | | | 35.3 | | |  | | 52 | | | 38.0 | |  | | | 10 | | | 28.6 |  | | | |  | |  | |  | | |  |
|  | Black | 54 | 12.9 | | |  | | | 28 | | | 14.7 | | |  | | 11 | | | 8.0 | |  | | | 3 | | | 8.6 |  | | | |  | |  | |  | | |  |
|  |  |  |  | | |  | | |  | | |  | | |  | |  | | |  | |  | | |  | | |  | *0.59* | | | | *0.65* | | *0.32* | | *0.47* | | |  |
|  | Number^c^ |  |  | | |  | | |  | | |  | | |  | |  | | |  | |  | | |  | | |  |  | | | |  | |  | |  | | |  |
|  | **N** | 572 | 100 | | |  | | | 214 | | | 100 | | |  | | 159 | | | 100.0 | |  | | | 48 | | | 100.0 |  | | | |  | |  | |  | | |  |
|  | 1 | 192 | 33.6 | | |  | | | 46 | | | 21.5 | | |  | | 44 | | | 27.7 | |  | | | 20 | | | 41.7 |  | | | |  | |  | |  | | |  |
|  | 2-3 | 130 | 22.7 | | |  | | | 56 | | | 26.2 | | |  | | 34 | | | 21.4 | |  | | | 12 | | | 25.0 |  | | | |  | |  | |  | | |  |
|  | >4 | 250 | 43.7 | | |  | | | 112 | | | 52.3 | | |  | | 81 | | | 50.9 | |  | | | 16 | | | 33.3 |  | | | |  | |  | |  | | |  |
|  |  |  |  | | |  | | |  | | |  | | |  | |  | | |  | |  | | |  | | |  | *0.005* | | | | *0.08* | | *0.24* | | *0.01* | | |  |
|  | Size^c^ |  |  | | |  | | |  | | |  | | |  | |  | | |  | |  | | |  | | |  |  | | | |  | |  | |  | | |  |
|  | **N** | 668 | 100 | | |  | | | 224 | | | 100 | | |  | | 196 | | | 100.0 | |  | | | 53 | | | 100.0 |  | | | |  | |  | |  | | |  |
|  | <1 cm | 229 | 34.3 | | |  | | | 61 | | | 25 | | |  | | 58 | | | 29.6 | |  | | | 17 | | | 32.1 |  | | | |  | |  | |  | | |  |
|  | 1-1.9 cm | 258 | 38.6 | | |  | | | 115 | | | 47.1 | | |  | | 84 | | | 42.9 | |  | | | 24 | | | 45.3 |  | | | |  | |  | |  | | |  |
|  | 2.0-2.9 cm | 115 | 17.3 | | |  | | | 43 | | | 17.6 | | |  | | 35 | | | 17.9 | |  | | | 10 | | | 18.9 |  | | | |  | |  | |  | | |  |
|  | >3.0 cm | 66 | 9.9 | | |  | | | 25 | | | 10.2 | | |  | | 19 | | | 9.7 | |  | | | 2 | | | 3.8 |  | | | |  | |  | |  | | |  |
|  |  |  |  | | |  | | |  | | |  | | |  | |  | | |  | |  | | |  | | |  | *0.05* | | | | *0.63* | | *0.63* | | *0.42* | | |  |
|  | Weight (g)^d^ |  |  | | |  | | |  | | |  | | |  | |  | | |  |  | | | |  | | |  |  | | | |  | |  | |  | | |  |
|  | N | 358 | 100 | | |  | | | 162 | | | 100 | | |  | | 41 | | | 100 |  | | | | 10 | | | 100 |  | | | |  | |  | |  | | |  |
|  | <1 | 103 | 28.8 | | |  | | | 58 | | | 35.8 | | |  | | 5 | | | 12.2 |  | | | | 5 | | | 50.0 |  | | | |  | |  | |  | | |  |
|  | 1-3 | 95 | 26.5 | | |  | | | 49 | | | 30.2 | | |  | | 13 | | | 31.7 |  | | | | 2 | | | 20.0 |  | | | |  | |  | |  | | |  |
|  | 3.1-4.9 | 94 | 26.3 | | |  | | | 36 | | | 22.2 | | |  | | 7 | | | 17.1 |  | | | | 1 | | | 10.0 |  | | | |  | |  | |  | | |  |
|  | > 5 | 66 | 18.4 | | |  | | | 19 | | | 11.7 | | |  | | 16 | | | 39.0 |  | | | | 2 | | | 20.0 |  | | | |  | |  | |  | | |  |
|  |  |  |  | | |  | | |  | | |  | | |  | |  | | |  | |  | | |  | | |  | *0.10* | | | | *0.09* | | *0.006* | | *0.58* | | |  |
|  | Average weight (g) (SD) | 2.8 | (2.6) | | |  | | | 2.3 | | | (2.4) | | |  | | 4.9 | | | (5.7) | |  | | | 2.2 | | | (2.8) | *0.92* | | | | *0.02* | | *0.01* | | *0.4* | | |  |
|  | Biochemical composition (mean)^e^ |  |  |  | | | |  | | |  | | |  | | | |  |  | | | |  |  | | | |  |  | | |  | | |  | | | |  | |
| N | | 358 |  |  | | | | 162 | | |  | | |  | | | | 41 |  | | | |  | 10 | | | |  |  | | |  | | |  | |  | | | |
| Cholesterol % (mg/100mg) | | 59.5 |  |  | | | | 53.9 | | |  | | |  | | | | 65.4 |  | | | |  | 55.2 | | | |  | *0.01* | | | *0.24* | | | *0.08* | | *0.88* | | | |
| Bilirubin % (mg/100mg) | | 4.3 |  |  | | | | 7.8 | | |  | | |  | | | | 3.9 |  | | | |  | 2.7 | | | |  | *<0.0001* | | | *0.28* | | | *0.62* | | *<0.0001* | | | |
| N | | 310 |  |  | | | | 125 | | |  | | |  | | | | 35 |  | | | |  | 9 | | | |  |  | | |  | | |  | |  | | | |
| Total bile acid (mg/100mg) | | 1.67 |  |  | | | | 3.85 | | |  | | |  | | | | 0.54 |  | | | |  | 1.52 | | | |  | *0.0005* | | | *0.36* | | | *0.0003* | | *0.02* | | | |
| Free bile acid (umol/100mg) | |  |  |  | | | |  | | |  | | |  | | | |  |  | | | |  |  | | | |  |  | | |  | | |  | |  | | | |
| Cholic acid | | 0.2 |  |  | | | | 1.56 | | |  | | |  | | | | 0.07 |  | | | |  | 0 | | | |  | *0.001* | | | *--* | | | *0.07* | | *--* | | | |
| Chenodeoxycholic acid | | 0.59 |  |  | | | | 3.38 | | |  | | |  | | | | 0.11 |  | | | |  | 0.09 | | | |  | *0.13* | | | *--* | | | *0.04* | | *--* | | | |
| Deoxycholic acid | | 0.27 |  |  | | | | 0.83 | | |  | | |  | | | | 0.02 |  | | | |  | 2.15 | | | |  | *0.61* | | | *0.50* | | | *0.01* | | *0.59* | | | |
| Glyco-conjugated bile acid | |  |  |  | | | |  | | |  | | |  | | | |  |  | | | |  |  | | | |  |  | | |  | | |  | |  | | | |
| Glycocholic acid | | 0.75 |  |  | | | | 1.27 | | |  | | |  | | | | 0.19 |  | | | |  | 0.64 | | | |  | *0.03* | | | *0.18* | | | *0.002* | | *0.04* | | | |
| Glychenodeocycholic acid | | 1.45 |  |  | | | | 1.56 | | |  | | |  | | | | 0.53 |  | | | |  | 0.54 | | | |  | *0.39* | | | *0.96* | | | *0.0009* | | *0.004* | | | |
| Clycodeoxycholic acid | | 0.4 |  |  | | | | 0.3 | | |  | | |  | | | | 0.23 |  | | | |  | 0.11 | | | |  | *0.47* | | | *0.25* | | | *0.28* | | *0.009* | | | |
|  | Glycolithocholic acid | 0.03 |  | |  | | | 0.1 | | |  | |  | | | | | 0.01 |  | | | |  | 0.02 | | |  | | | *0.22* | | | | *0.21* | | *0.54* | | *0.14* | | |
|  | ^a^ Based on Chi-square or exact test for categorical variables and t-test for continuous variables  ^b^ p1: Comparison between gallstone and bile duct stone cases  p2: Comparison between gallbladder and bile duct cancer cases  p3: Comparison between gallbladder cancer and gallstone cases  p4: Comparison between bile duct cancer and bile duct stone cases | | | | | | | | | | | | | | | | | | | | | | | | | | | | | | | | | | | | | | | |
|  | ^c^Based on information from medical records. | | | | | | | | | | | | | | | | | | | | | | | | | | | | | | | | | | | | | | | |
|  | ^d^Based on all stones collected.  ^e^Based on the largest stone collected. | | | | | | | | | | | | | | | | | | | | | | | | | | | | | | | | | | | | | | | |
